# Dose–response relationship of physical exercise interventions for balance performance in children and adolescents with intellectual disabilities: a systematic review and meta-analysis

**DOI:** 10.3389/fpubh.2025.1686892

**Published:** 2025-09-25

**Authors:** Ziqi Gao, Yafan Li, Yaxin Wang, Wei Quan, Hongnan Meng, Jianxin Kang

**Affiliations:** ^1^Postgraduate School, Harbin Sport University, Harbin, China; ^2^School of Physical Education, Heilongjiang University of Technology, Jixi, China

**Keywords:** exercise intervention, intellectual disability, children and adolescents, balance performance, dose–response relationship, meta-analysis

## Abstract

**Objective:**

To systematically evaluate the impact of exercise interventions on balance performance in children and adolescents with intellectual disabilities and examine the dose–response relationship of key intervention parameters.

**Methods:**

A comprehensive search of PubMed, Web of Science, Embase, Scopus, and the Cochrane Library identified eligible randomized controlled trials (RCTs). Standardized mean differences (SMDs) with 95% confidence intervals (CIs) were calculated using a random-effects model. Subgroup analyses and non-linear meta-regression were performed to explore potential effect modifiers and dose–response patterns.

**Results:**

A total of 23 eligible studies comprising 31 datasets and 1,179 participants were included. Pooled analysis showed significant improvement in balance performance [SMD = 0.69, 95% CI (0.48, 0.89), *p* < 0.001]. Dose–response modeling indicated maximal benefit at approximately 717 MET·min/week (Hedges' *g* = 0.76). Subgroup analyses revealed greater effects with a frequency of ≥3 sessions/week [SMD = 0.76, 95% CI (0.48, 1.03), *p* < 0.001], session duration >60 min [SMD = 0.82, 95% CI (0.35, 1.29), *p* = 0.001], and intervention period < 8 weeks [SMD = 0.78, 95% CI (0.46, 1.10), *p* < 0.001].

**Conclusion:**

Moderate-dose exercise (717 MET·min/week) can significantly improve the balance performance in children and adolescents with intellectual disabilities, with specific frequency, duration, and intervention period combinations yielding superior outcomes. These findings provide an evidence-based basis for precision health strategies. However, moderate heterogeneity highlights the need for confirmation through larger, multicenter, and long-term trials.

## Introduction

Intellectual disability (ID) is a neurodevelopmental disorder with onset during the developmental period, characterized by significant deficits in intellectual functioning and adaptive behavior, which persistently impair an individual's independence and social participation (1). According to the World Health Organization (WHO), approximately 1% of children and adolescents worldwide are affected, with mild and moderate forms accounting for the majority of cases (2). This population often presents with delays in motor development and deficits in fundamental motor skills (3), particularly in balance control and postural stability (4).

Balance performance is a fundamental motor skill underpinning both daily activities and sports participation, encompassing dynamic balance, static balance, and the integrated ability to transition between these states (5). It depends on the coordinated integration of multiple sensory systems and neuromuscular functions, including the visual, vestibular, and proprioceptive systems (6–8). Impaired balance not only increases the risk of falls and injuries but may also undermine self-confidence and social engagement, thereby reducing overall quality of life (9, 10). Improving balance performance is therefore of particular importance for children and adolescents with ID.

In recent years, exercise interventions have emerged as a non-pharmacological strategy to enhance both motor and cognitive functions in this population (11). Evidence suggests that balance training, gait training, and core stability exercises can effectively improve balance performance in children and adolescents with ID (12, 13). However, most available studies are limited by small sample sizes, and few have systematically compared or synthesized the effects of different intervention protocols. The optimal regimen and its underlying dose–response relationship remain unclear. Furthermore, intervention outcomes may be moderated by factors such as intervention type, frequency, duration, total length, the specific dimension of balance targeted, and the severity of intellectual disability.

The present study employs a meta-analytic approach to evaluate the effects of exercise interventions on balance performance in children and adolescents with ID. Specifically, it quantifies the effect sizes of different intervention types, examines the moderating roles of key exercise prescription parameters (e.g., frequency, session duration, intervention length), and explores potential dose–response relationships. In addition, it investigates the presence and sources of between-study heterogeneity. The ultimate aim is to provide an evidence-based foundation for the individualized optimization of exercise interventions, thereby advancing precision health strategies for individuals with ID.

## Methods

This systematic review was conducted in strict accordance with the Preferred Reporting Items for Systematic Reviews and Meta-Analyses (PRISMA) guidelines (14) and was prospectively registered in the PROSPERO database (registration number: CRD420251105005).

### Search strategy

A comprehensive literature search was performed in PubMed, Embase, Scopus, Web of Science, and the Cochrane Library from database inception to July 25, 2025. The search was limited to English-language publications and randomized controlled trials (RCTs). A combination of Medical Subject Headings (MeSH) and free-text terms was used, covering four key domains: “intellectual disability,” “exercise intervention,” “children and adolescents,” and “balance performance.” The complete search strategies for each database are provided in Supplementary material A. Two reviewers independently and blindly screened the search results, and any discrepancies were resolved through discussion with a third reviewer.

### Inclusion criteria

(1) Participants were children or adolescents (aged 6–18 years) with a confirmed diagnosis of intellectual disability.
(2) Published in English.
(3) Designed as a randomized controlled trial.
(4) The intervention consisted of regular, structured exercise programs (e.g., aerobic exercise, balance training, strength training).
(5) The control group did not receive any structured exercise intervention and participated only in routine lifestyle activities or standard school-based physical education.
(6) Reported at least one objective, quantitative outcome measure of balance performance (static, dynamic, or combined static–dynamic balance) assessed using standardized tools such as the Berg Balance Scale, Stork Balance Test, or Bruininks–Oseretsky Test of Motor Proficiency, Second Edition (BOT-2).

### Exclusion criteria

(1) Animal experiments or non-human studies.
(2) Duplicate publications, low-quality study designs, or a high risk of bias.
(3) Full text unavailable or insufficient data to extract balance-related outcomes.
(4) Did not employ objective balance-related measurement tools as outcomes.
(5) Non-RCT study types, including reviews, systematic reviews, meta-analyses, conference abstracts, commentaries, or secondary data analyses.
(6) Participants with specific genetic syndromes associated with intellectual disability (e.g., Down syndrome, Prader–Willi syndrome, Williams syndrome) or with severe physical or psychiatric comorbidities (e.g., cerebral palsy, autism spectrum disorder, severe motor impairment) that could confound balance performance assessment.

### Study selection and data extraction

Two reviewers independently conducted the processes of study selection, data extraction, and quality assessment, with any disagreements resolved by a third reviewer. All retrieved records were imported into EndNote 21, and duplicates were removed. Titles and abstracts were screened against the inclusion and exclusion criteria for preliminary eligibility, followed by full-text review of potentially relevant articles to confirm inclusion. For each eligible study, the following information was extracted:

(1) Study characteristics: first author, year of publication, and journal.
(2) Participant and intervention details: sample size, age, intervention duration, session length, and frequency for both experimental and control groups.
(3) Outcome measures: static balance, dynamic balance, and combined static–dynamic balance.

When outcome data were missing or incomplete, attempts were made to contact the study authors to obtain the relevant information.

### Quality assessment

The methodological quality of the included studies was evaluated using the Cochrane Risk of Bias (RoB) tool, assessing seven domains: random sequence generation, allocation concealment, blinding of participants and personnel, blinding of outcome assessment, incomplete outcome data, selective reporting, and other potential sources of bias. Risk-of-bias graphs were generated in Review Manager (RevMan) version 5.3. The quality assessment results were subsequently used to inform sensitivity analyses and to aid in interpreting potential sources of heterogeneity.

### Data analysis

All statistical analyses were performed using Stata version 18. As the included outcomes were continuous variables, weighted mean differences with 95% confidence intervals (CIs) were calculated for studies using the same measurement tools, whereas standardized mean differences (SMDs) were used for studies employing different tools. Heterogeneity was quantified using *p*-values and the *I*^2^ statistic, with *I*^2^ values ranging from 0 to 100%. An *I*^2^ < 50% with *p* > 0.1 was considered indicative of low heterogeneity, and a fixed-effects model was applied; an *I*^2^ ≥ 50% with *p* < 0.1 indicated substantial heterogeneity, for which a random-effects model was used.

Potential moderators contributing to heterogeneity were explored through subgroup analyses and meta-regression. Statistical significance was set at *p* < 0.05. Publication bias was assessed visually via funnel plots, and sensitivity analyses were performed using a leave-one-out approach.

To further examine the relationship between exercise dosage and intervention effects, the weekly total dose for each intervention was calculated in MET-min/week based on the Compendium of Physical Activities (15), standardizing intensity, frequency, and duration across studies. A non-linear dose–response analysis was then conducted using restricted cubic splines within a random-effects model (16).

## Results

### Search results

**Figure 1:**
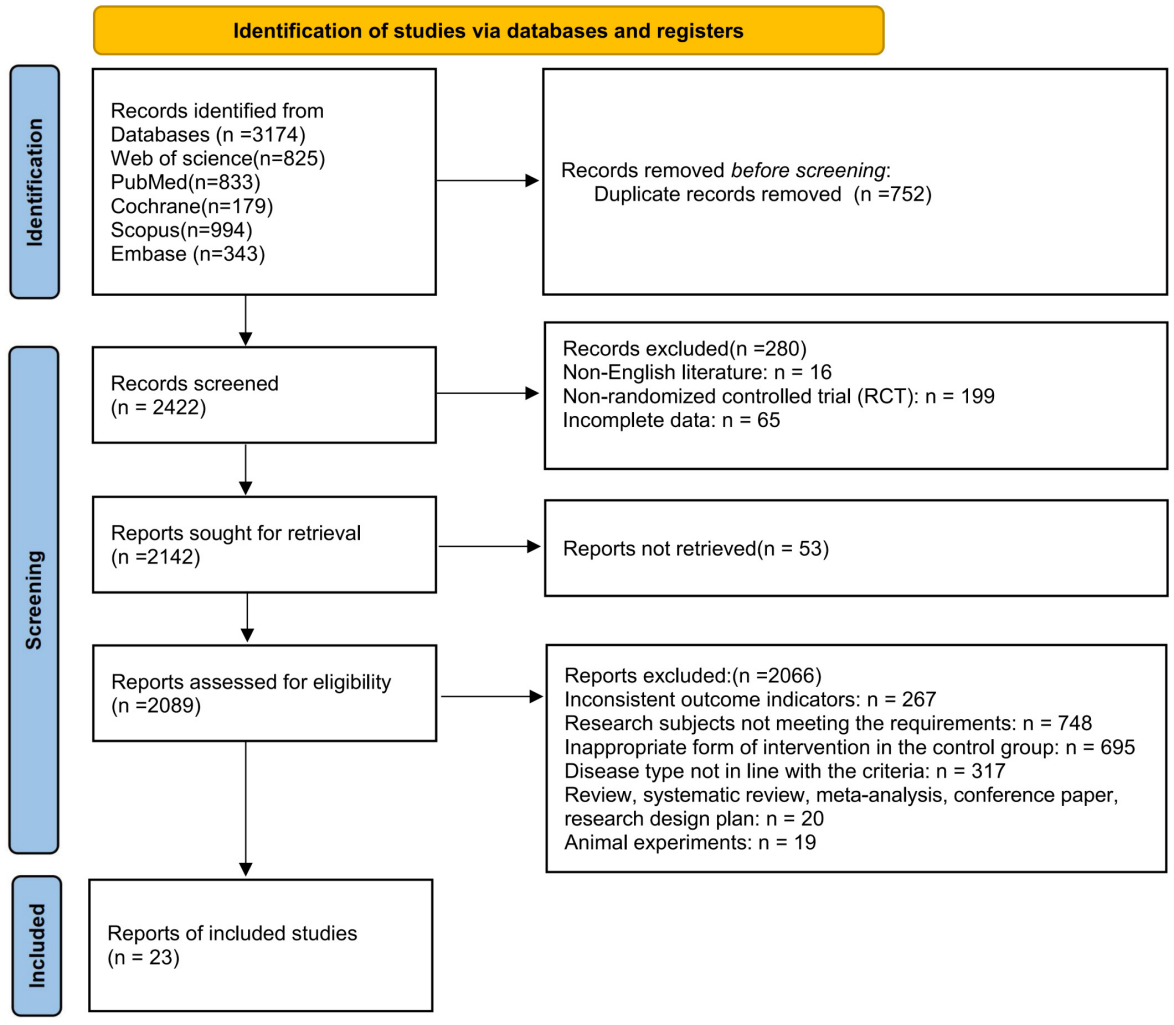
PRISMA flow diagram of study selection.

A total of 3,174 records were identified through the PubMed, Embase, Scopus, Web of Science, and Cochrane Library databases. The PRISMA flow diagram summarizing the study selection process is presented in Figure 1. After removing duplicates, screening titles and abstracts, and excluding studies not meeting the eligibility criteria, 23 articles were included (17–39), comprising 31 randomized controlled trials (RCTs).

### Characteristics of included studies

The included studies involved a total of 1,179 children and adolescents with intellectual disabilities (607 in the intervention groups and 572 in the control groups), with a mean age of 13.48 ± 1.37 years. Intervention groups received aerobic exercise, strength training, or balance training, whereas control groups maintained a routine lifestyle activities or participated in standard school physical education.

Exercise intervention parameters primarily included intervention duration, session length, and frequency. The intervention period ranged from 6 to 52 weeks, session duration from 15 to 90 min, and frequency from 2 to 5 sessions per week. Balance-related outcomes encompassed static balance, dynamic balance, and combined static–dynamic balance, measured using tools such as the Stork Balance Test, force platform postural sway test, EPS pressure platform, Berg Balance Scale, Bruininks–Oseretsky Test of Motor Proficiency–Short Form (BOT-2 SF), Timed Up-and-Go Test (TUG), and the Y Balance Test.

For the included balance assessments, higher scores or smaller sway range (cm), shorter total path length (cm), and smaller platform sway area (cm^2^) indicated better balance performance (Table 1).

**Table 1:** Characteristics of included studies.

**First author**	**Country**	**Experimental group**	**Control group**	**Age (years)**	**Intellectual disability severity**	**Experimental group**	**Control group**	**Intervention duration (weeks)**	**Session duration (min/ session)**	**Frequency (sessions/ week)**	**Outcome indicator**
Ahmadi et al. (17)	Iran	17	14	11.50 ± 2.02	Mild	Strength training	Regular lifestyle	6	45	3	Static balance
Farrokhian et al. (22)	Iran	15	15	9.50 ± 2.02	Mild	Strength training	Regular lifestyle	5	60	3	Dynamic balance
Fotiadou et al. (23)	Greece	10	10	10.00 ± 1.15	Moderate	Aerobic exercise	Regular lifestyle	16	45	2	Static balance
Mehralitabar et al. (34)	Iran	12	12	10.50 ± 1.44	Mild	Aerobic exercise	Regular lifestyle	8	30	3	Dynamic-Static balance
Zolghadr et al. (39)	Iran	12	11	16.00 ± 1.15	Mild	Balance training	Regular lifestyle	6	45	3	Static balance
Zolghadr et al. (39)	Iran	12	11	16.00 ± 1.15	Mild	Balance training	Regular lifestyle	6	60	3	Dynamic balance
Kachouri et al. (31)	Tunisia	10	10	11.00 ± 1.15	Mild	Strength training	School sports	8	60	3	Static balance
Lee et al. (33)	South Korea	15	16	16.50 ± 1.44	Mild	Balance training	Regular lifestyle	24	45	3	Dynamic Balance
Dehghani et al. (21)	Turkey	11	11	10.00 ± 1.15	Mild	Aerobic exercise	School sports	10	40	2	Dynamic balance
Dehghani et al. (21)	Turkey	11	11	10.00 ± 1.15	Mild	Aerobic exercise	School sports	10	40	2	Dynamic-Static balance
Işik et al. (29)	Turkey	25	25	14.00 ± 1.15	Moderate	Aerobic exercise	School sports	12	60	3	Dynamic-Static balance
Kubilay et al. (32)	Turkey	14	14	15.50 ± 3.75	Mild	Aerobic Exercise	Regular Lifestyle	8	55	3	Dynamic-Static Balance
Giagazoglou et al. (24)	Greece	10	19	15.30 ± 1.21	Moderate	Aerobic exercise	Regular lifestyle	10	30	2	Static balance
Giagazoglou et al. (25)	Greece	10	19	10.30 ± 0.92	Mild	Aerobic exercise	Regular lifestyle	12	20	5	Static balance
Rahmat et al. (36)	Iran	17	14	11.50 ± 2.02	Mild	Strength training	School sports	6	45	4	Dynamic balance
Borji et al. (20)	Tunisia	10	10	11.75 ± 0.90	Mild	Aerobic Exercise	Regular Lifestyle	8	40	2	Dynamic-Static Balance
Golubovic et al. (26)	Serbia	21	21	9.25 ± 1.59	Mild	Aerobic exercise	Regular lifestyle	6	45	3	Dynamic-Static balance
Jankowicz-Szymanska et al. (30)	Poland	20	20	17.00 ± 0.58	Moderate	Aerobic exercise	Regular lifestyle	10	45	2	Static balance
Wu et al. (37)	Taiwan, China	14	14	16.00 ± 1.73	Moderate	Aerobic exercise	School sports	8	60	3	Static balance
Wu et al. (37)	Taiwan, China	14	14	16.00 ± 1.73	Moderate	Aerobic exercise	School sports	8	60	3	Dynamic balance
Zhao et al. (38)	China	14	14	10.61 ± 0.10	Moderate	Balance Training	Regular Lifestyle	12	60	3	Static Balance
Aslan et al. (18)	Turkey	65	48	16.21 ± 1.71	Mild	Aerobic exercise	Regular lifestyle	52	60	2	Static balance
Aslan et al. (18)	Turkey	45	39	16.93 ± 1.72	Moderate	Aerobic exercise	Regular lifestyle	52	60	2	Static balance
Aslan et al. (18)	Turkey	65	48	16.21 ± 1.71	Mild	Aerobic exercise	Regular lifestyle	52	40	2	Dynamic balance
Aslan et al. (18)	Turkey	45	39	16.93 ± 1.72	Moderate	Aerobic exercise	Regular lifestyle	52	40	2	Dynamic balance
Azimizadeh et al. (19)	Iran	12	12	16.27 ± 1.17	Mild	Balance training	Regular lifestyle	8	40	2	Static balance
Azimizadeh et al. (19)	Iran	12	12	11.00 ± 1.15	Mild	Strength training	Regular lifestyle	8	60	3	Dynamic balance
Mikolajczyk et al. (35)	Poland	17	17	15.06 ± 0.91	Moderate	Balance training	School sports	12	15	3	Dynamic balance
Hsu et al. (27)	Taiwan, China	27	27	16.59 ± 0.56	Mild	Aerobic exercise	Regular lifestyle	12	90	3	Dynamic-Static balance
Ilbeigi et al. (28)	Iran	10	10	12.2 ± 1.10	Mild	Aerobic exercise	Regular lifestyle	8	45	3	Static balance
Ilbeigi et al. (28)	Iran	15	15	12.20 ± 1.10	Mild	Aerobic training	Regular lifestyle	8	45	3	Dynamic balance

### Quality assessment results

**Figure 2:**
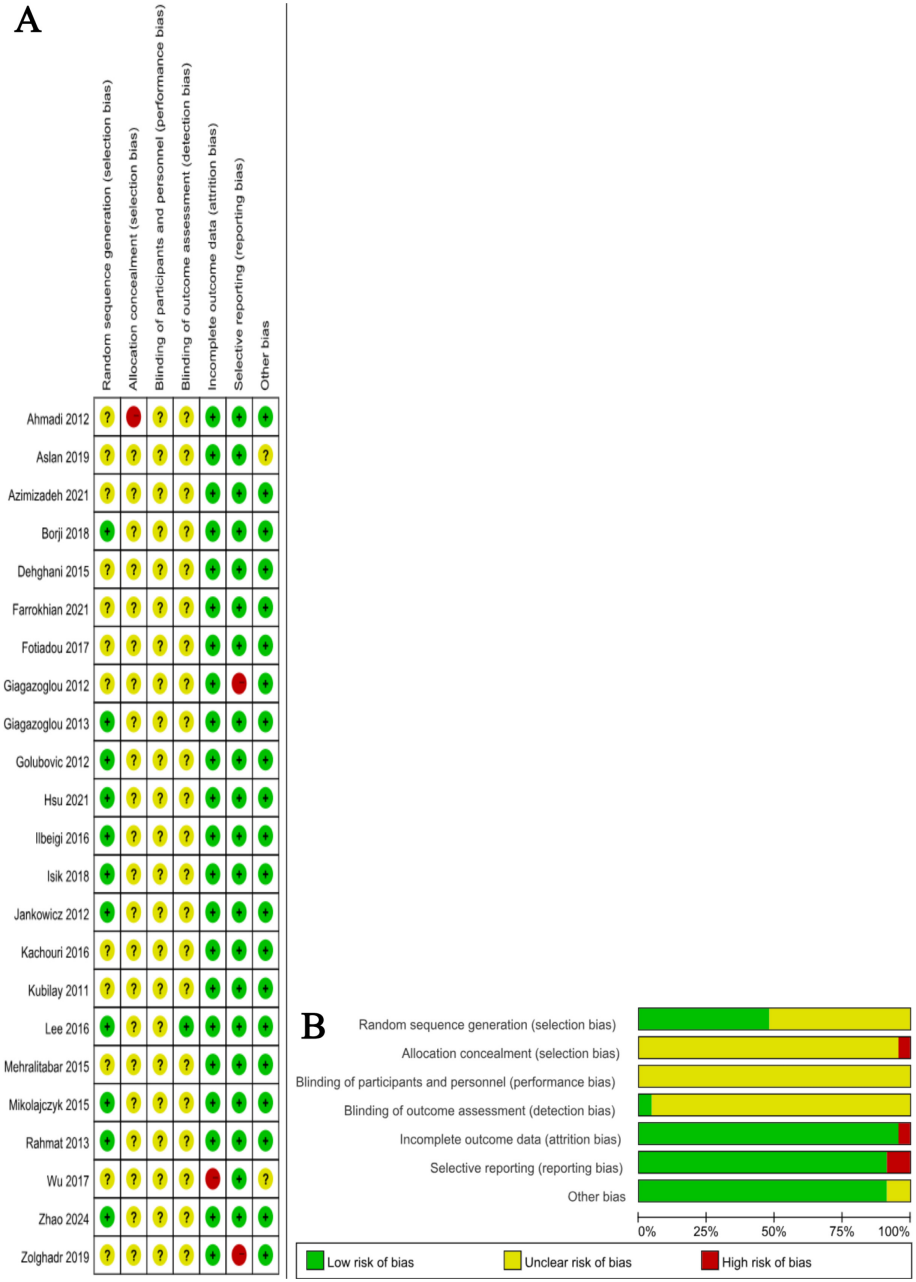
**(A)** Distribution of methodological quality; **(B)** Risk-of-bias graph and summary.

All 23 included studies were evaluated using the Cochrane Risk of Bias (RoB) assessment tool. The results indicated that most studies presented a low risk of bias in the domains of random sequence generation and completeness of outcome data, suggesting generally high methodological quality. However, in the domains of allocation concealment and blinding of participants and personnel, several studies were rated as having an unclear risk due to insufficient reporting of methodological details. A few studies demonstrated a high risk of bias in blinding of outcome assessment or selective reporting. Overall, the methodological quality of the included studies was moderate to high, with the risk of bias considered acceptable (Figure 2).

### Meta-analysis results

#### Effect of exercise interventions on balance performance

**Figure 3:**
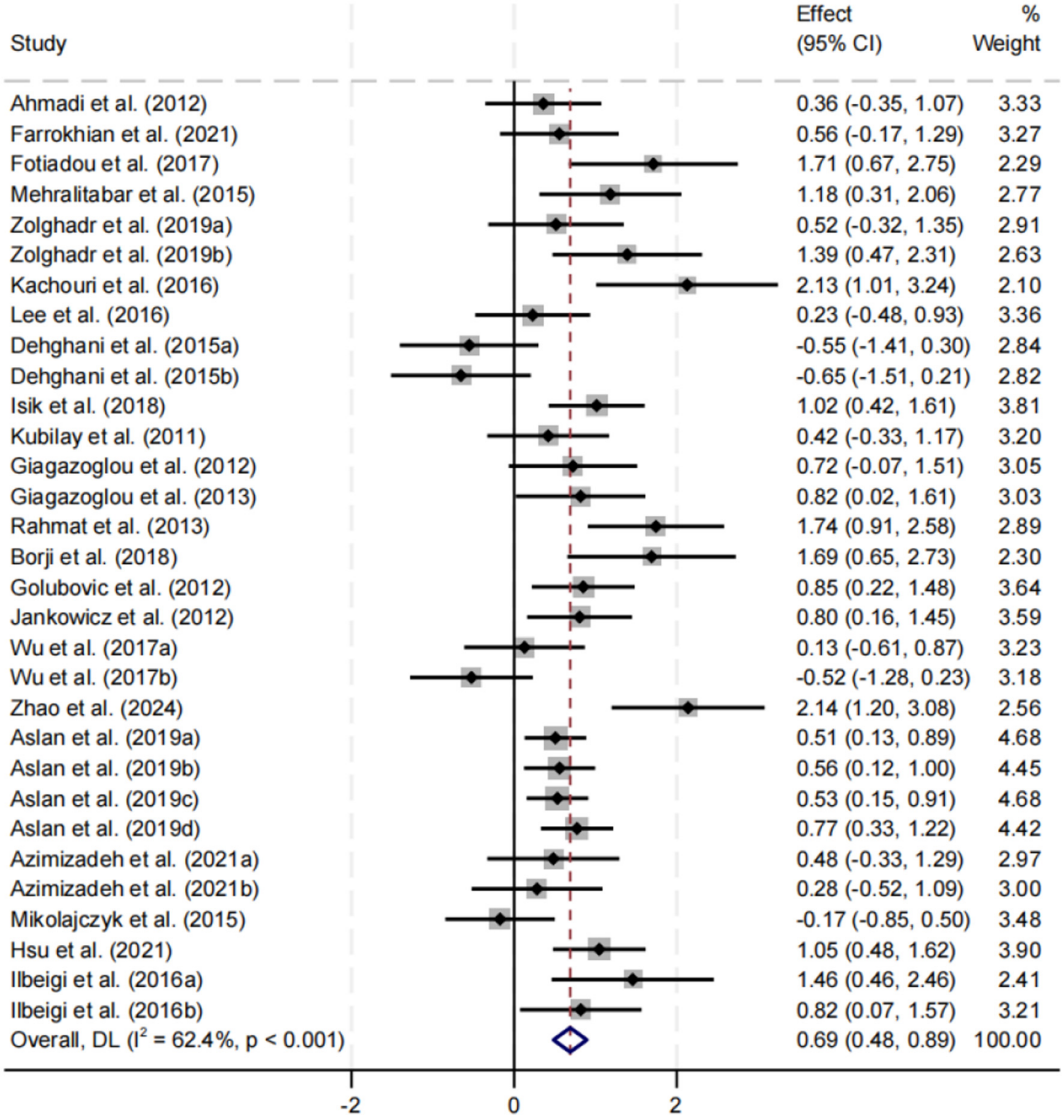
Forest plot of the effect of exercise interventions on balance performance.

A total of 31 datasets assessed the effects of exercise interventions on balance performance in children and adolescents with intellectual disabilities (Figure 3). Given the presence of moderate heterogeneity among studies (*I*^2^ = 62.4%, *p* < 0.001), a random-effects model was applied. The pooled analysis indicated that exercise interventions significantly improved balance performance compared with control conditions, yielding an overall effect size of SMD = 0.69 (95% CI: 0.48–0.89, *p* < 0.001).

#### Publication bias

**Figure 4:**
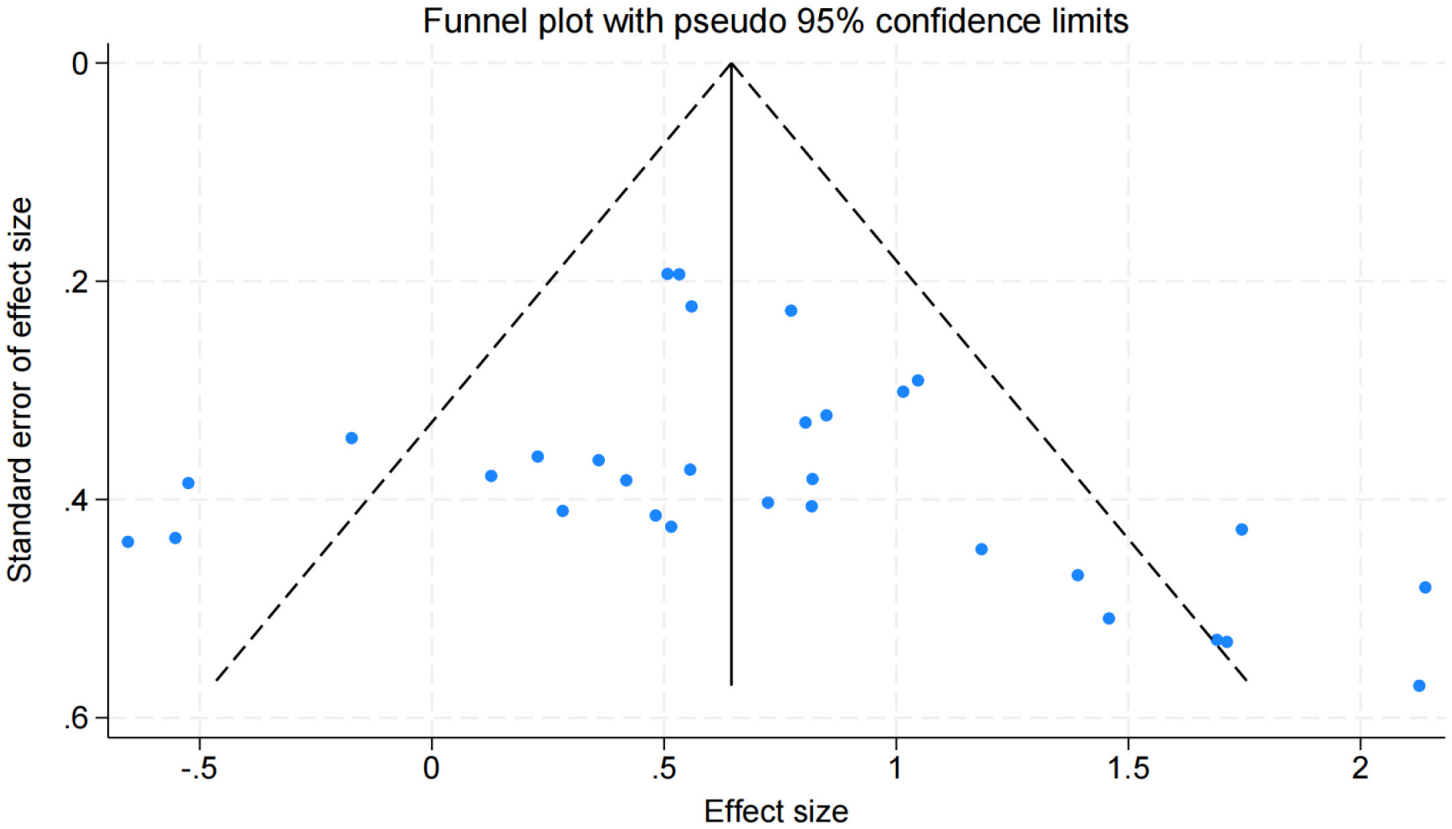
Funnel plot assessing publication bias for the effects of exercise interventions on balance performance.

Publication bias was preliminarily assessed using a funnel plot (Figure 4). The majority of studies were distributed symmetrically on both sides of the overall effect size, with the plot narrowing progressively from bottom to top, indicating an approximately symmetrical pattern. These results suggest a relatively low risk of publication bias in this meta-analysis.

#### Dose–response relationship analysis

**Figure 5:**
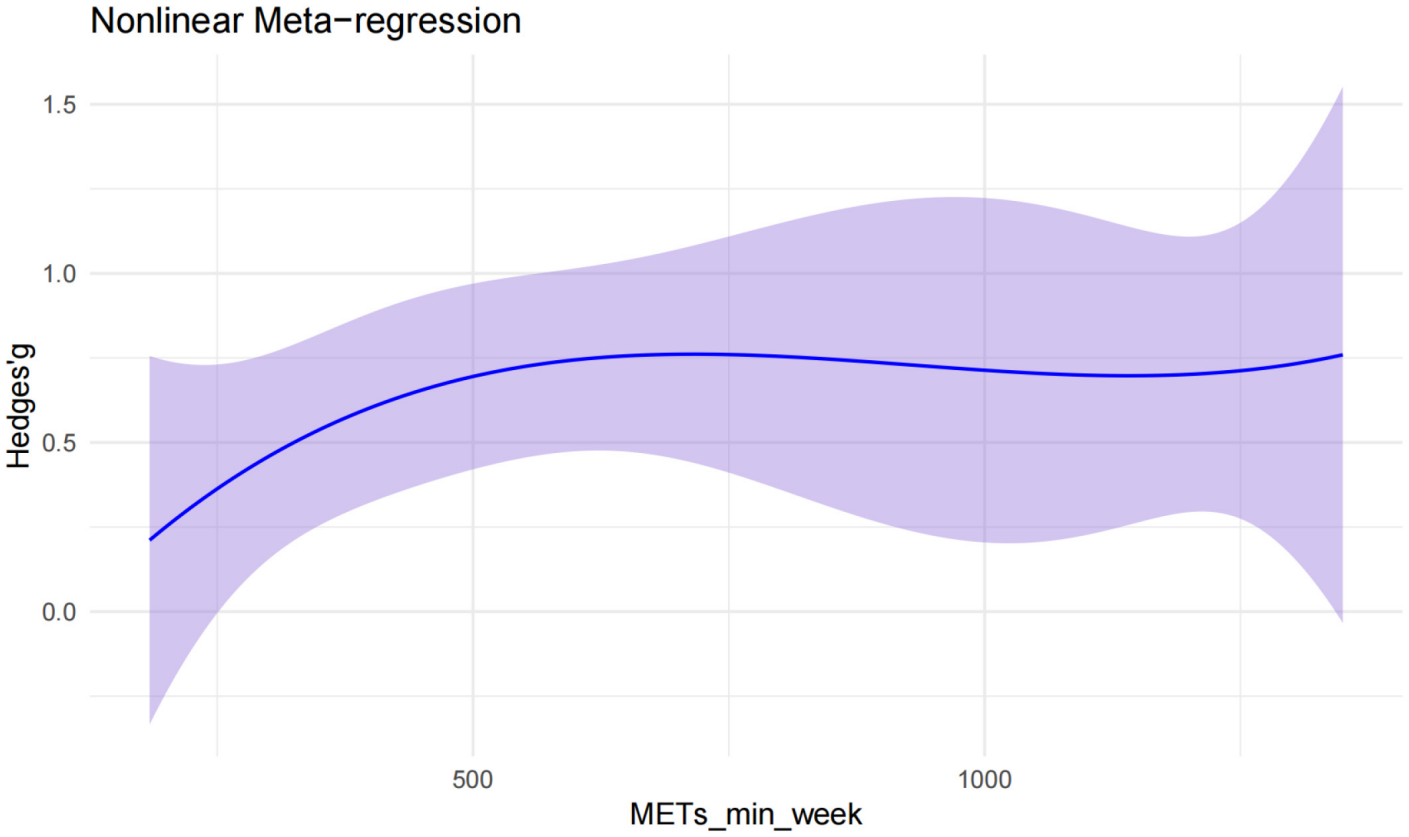
Non-linear dose–response relationship between exercise interventions and balance performance.

Figure 5 illustrates the non-linear dose–response relationship between weekly exercise volume and balance performance in children and adolescents with intellectual disabilities. To ensure the stability of estimates in the context of smaller sample sizes within the dose–response modeling, Hedges' *g* was used as the effect size metric. The analysis revealed that the peak intervention effect occurred at approximately 717 MET·min/week (Hedges' *g* = 0.76, 95% CI: 0.44–1.08).

At low exercise volumes (0–500 MET·min/week), the effect size increased rapidly with dosage. Between 600 and 800 MET·min/week, the growth in effect began to plateau, reaching a stable phase. When exercise volume exceeded 1,000 MET·min/week, the intervention effect remained relatively constant at around Hedges' *g* ≈ 0.7, indicating that additional increases in exercise dosage yielded minimal further gains. The non-linear model thus demonstrated a pattern of rapid initial improvement, followed by gradual growth and eventual stabilization of benefits.

#### Subgroup analyses

##### Effect of intellectual disability severity on balance performance

**Figure 6:**
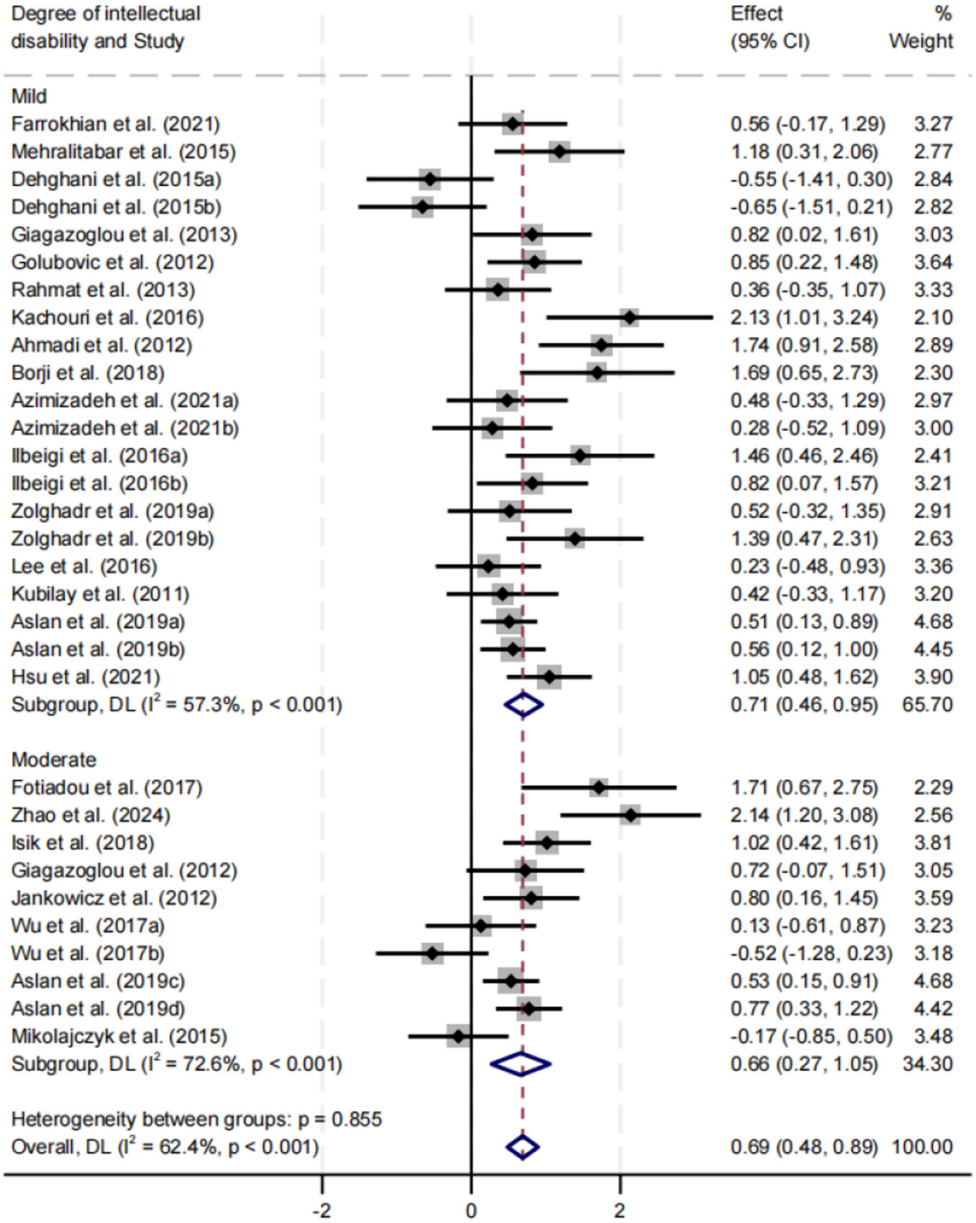
Subgroup analysis of the effect of intellectual disability severity on balance performance.

Subgroup analysis was performed for participants with mild and moderate intellectual disability (Figure 6). Both groups demonstrated significant improvements in balance performance, with a slightly larger effect observed in the mild group (SMD = 0.71). Heterogeneity was moderate in the mild group (*I*^2^ = 57.3%) and high in the moderate group (*I*^2^ = 72.6%), with both results statistically significant (*p* < 0.05). No significant difference was found between subgroups (*p* = 0.855).

##### Effect of exercise interventions on different types of balance performance

**Figure 7:**
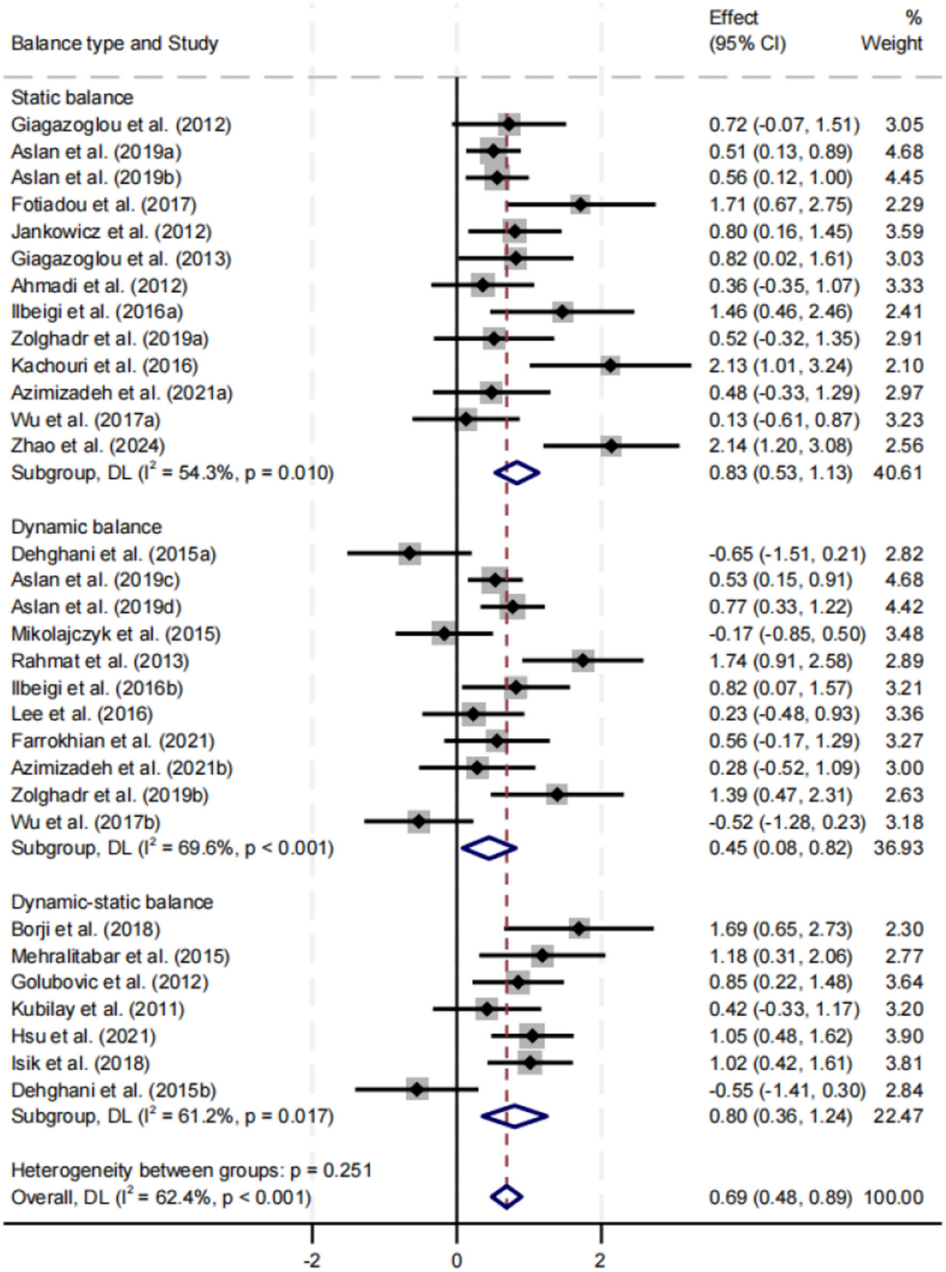
Subgroup analysis of the effect of exercise interventions on different types of balance performance.

Subgroup analysis was conducted for static balance, dynamic balance, and combined static–dynamic balance (Figure 7). All three types showed significant improvement, with SMDs of 0.83, 0.45, and 0.80, respectively (*p* < 0.05). The improvements in static and combined static–dynamic balance were comparable and greater than those observed for dynamic balance. Heterogeneity was highest for dynamic balance (*I*^2^ = 69.6%). Differences between subgroups were not statistically significant (*p* = 0.251).

##### Effect of motion type on balance performance

**Figure 8:**
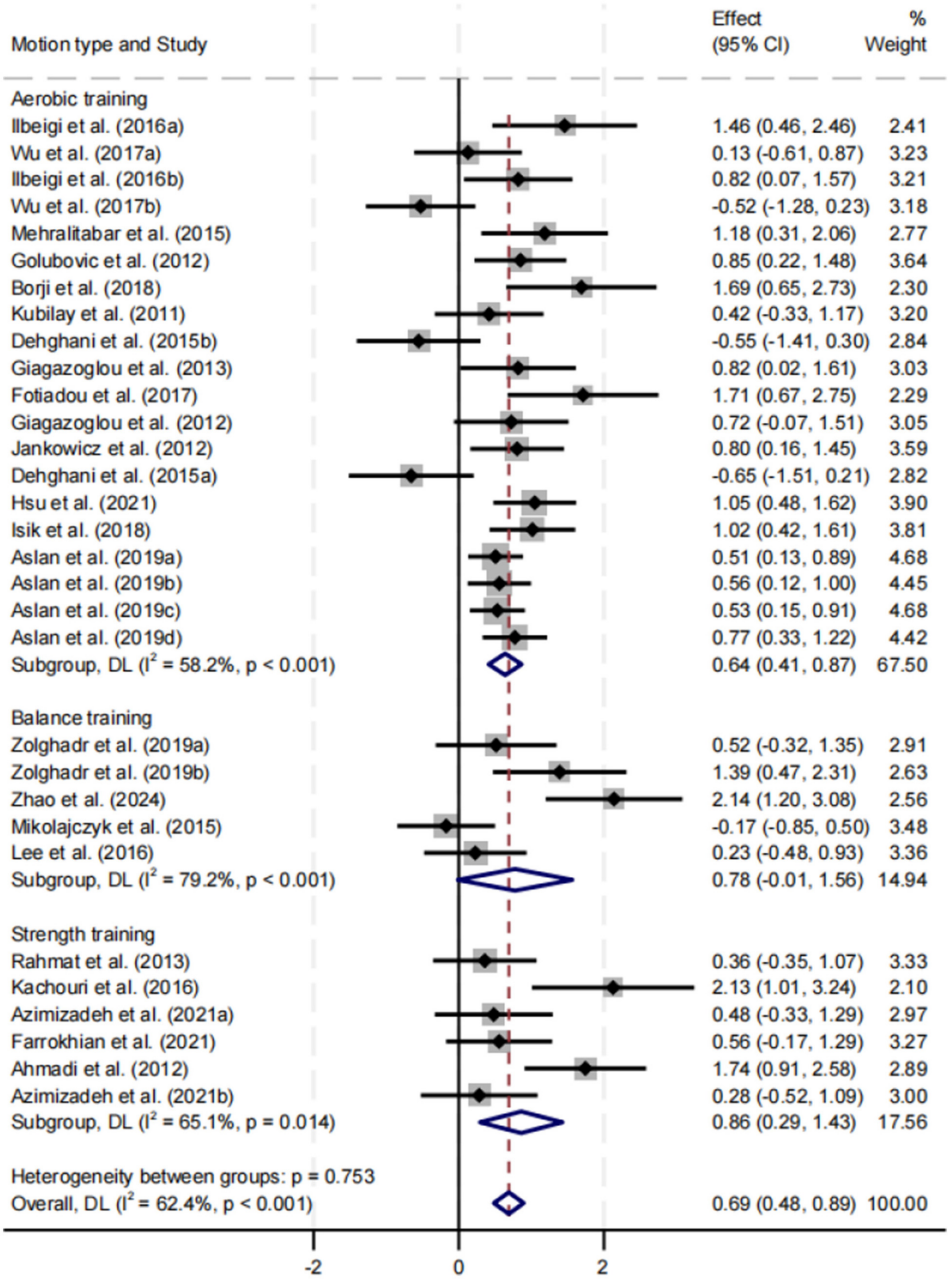
Subgroup analysis of the effect of motion type on balance performance.

Subgroup analysis was stratified by aerobic training, balance training, and strength training (Figure 8). All three intervention types significantly improved balance performance, with SMDs of 0.64, 0.78, and 0.86, respectively (*p* < 0.05). Strength training yielded the largest effect size, while balance training exhibited the highest heterogeneity (*I*^2^ = 79.2%). No statistically significant differences were found between subgroups (*p* = 0.753).

##### Effect of intervention duration on balance performance

**Figure 9:**
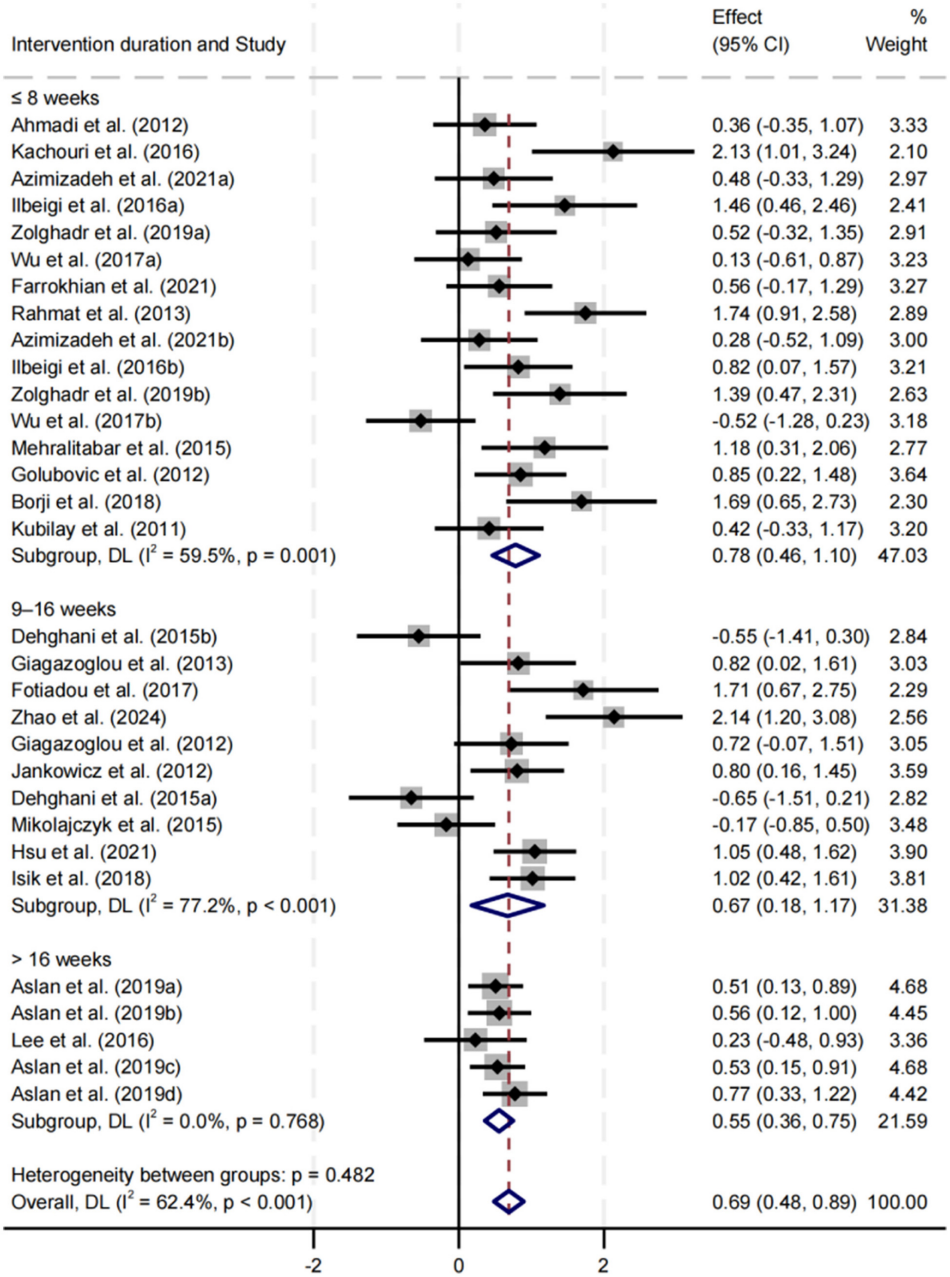
Subgroup analysis of the effect of intervention duration on balance performance.

Subgroup analysis was performed for intervention durations of ≤ 8, 9–16, and >16 weeks (Figure 9). All three durations significantly improved balance performance, with SMDs of 0.78, 0.67, and 0.55, respectively (*p* < 0.05). The ≤ 8 weeks group demonstrated the greatest improvement, with moderate heterogeneity (*I*^2^ = 59.5%, *p* = 0.001). No significant difference was found between subgroups (*p* = 0.482).

##### Effect of session length on balance performance

**Figure 10:**
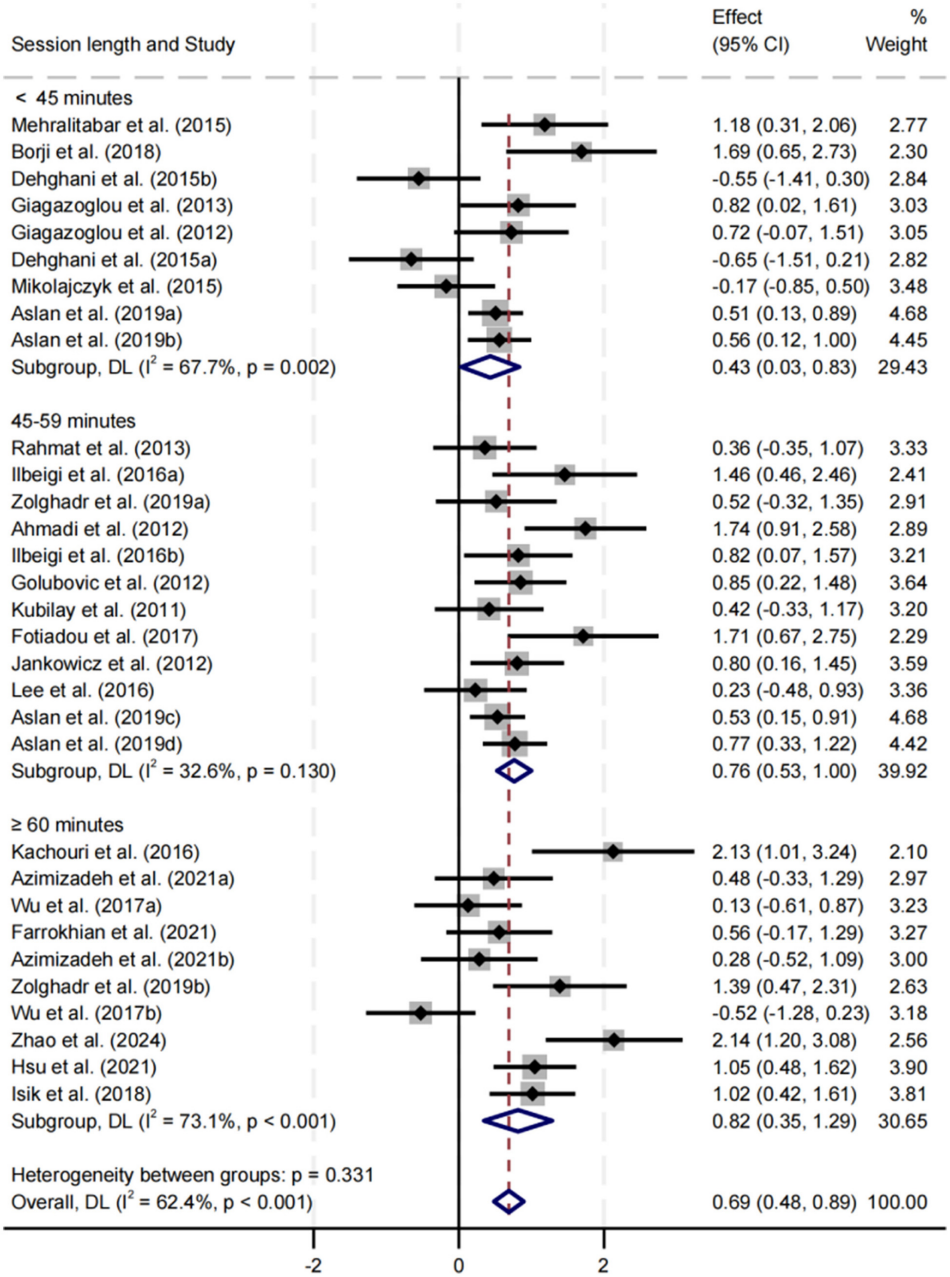
Subgroup analysis of the effect of session length on balance performance.

Subgroup analysis was conducted for session lengths of < 45, 45–59, and ≥60 min (Figure 10). All three durations significantly improved balance performance, with SMDs of 0.43, 0.76, and 0.82, respectively (*p* < 0.05). The ≥60 min group had the largest effect size, while the 45–59 min group exhibited the lowest heterogeneity (*I*^2^ = 32.6%). Differences between subgroups were not statistically significant (*p* = 0.331).

##### Effect of training frequency on balance performance

**Figure 11:**
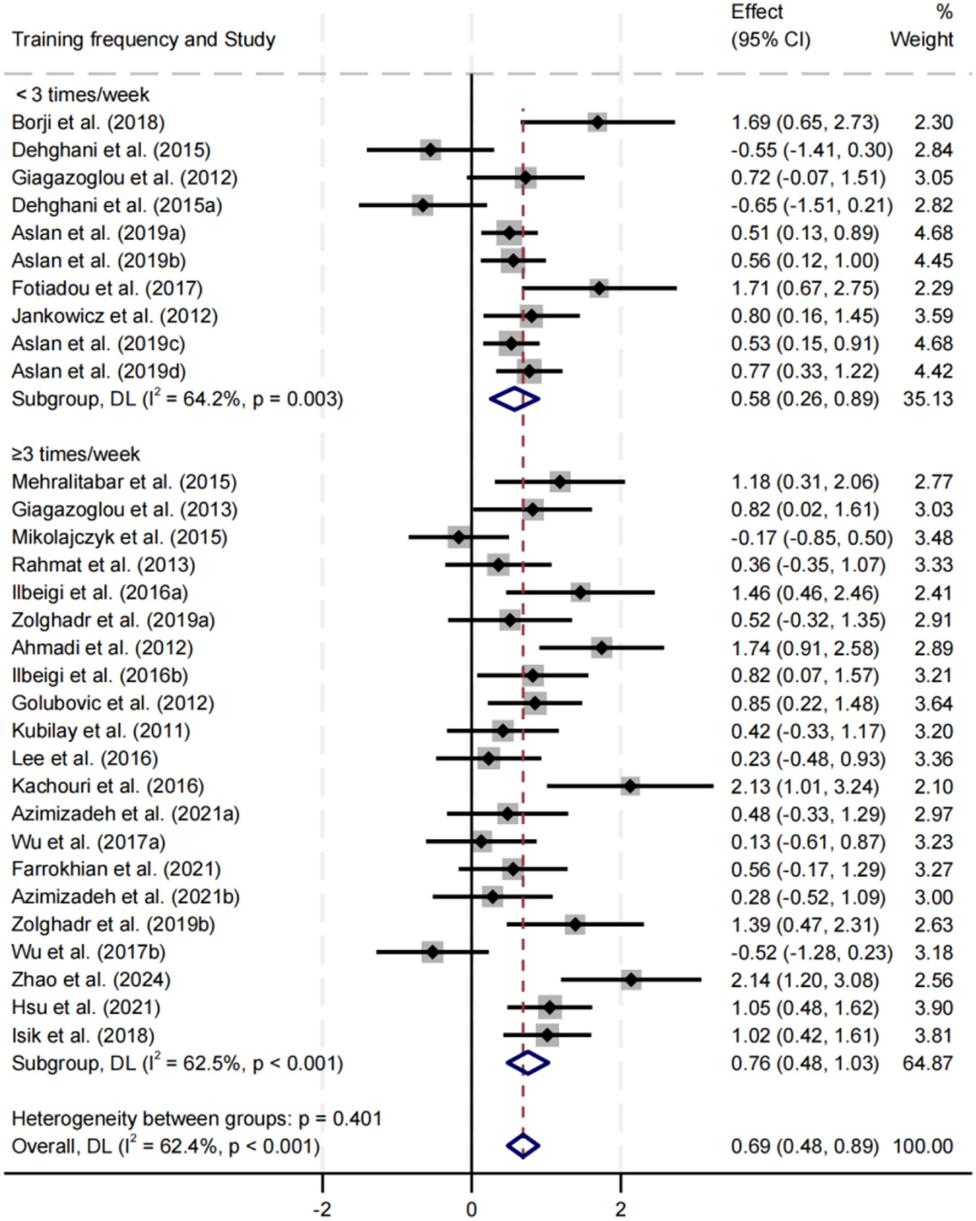
Subgroup analysis of the effect of training frequency on balance performance.

Subgroup analysis was conducted for training frequencies of < 3 sessions/week and ≥3 sessions/week (Figure 11). Both frequencies significantly improved balance performance, with SMDs of 0.58 and 0.76, respectively (*p* < 0.05). The ≥3 sessions/week group had a slightly higher effect size, and heterogeneity in both groups was moderate. No significant difference was observed between subgroups (*p* = 0.401).

#### Meta-regression analysis

**Figure 12:**
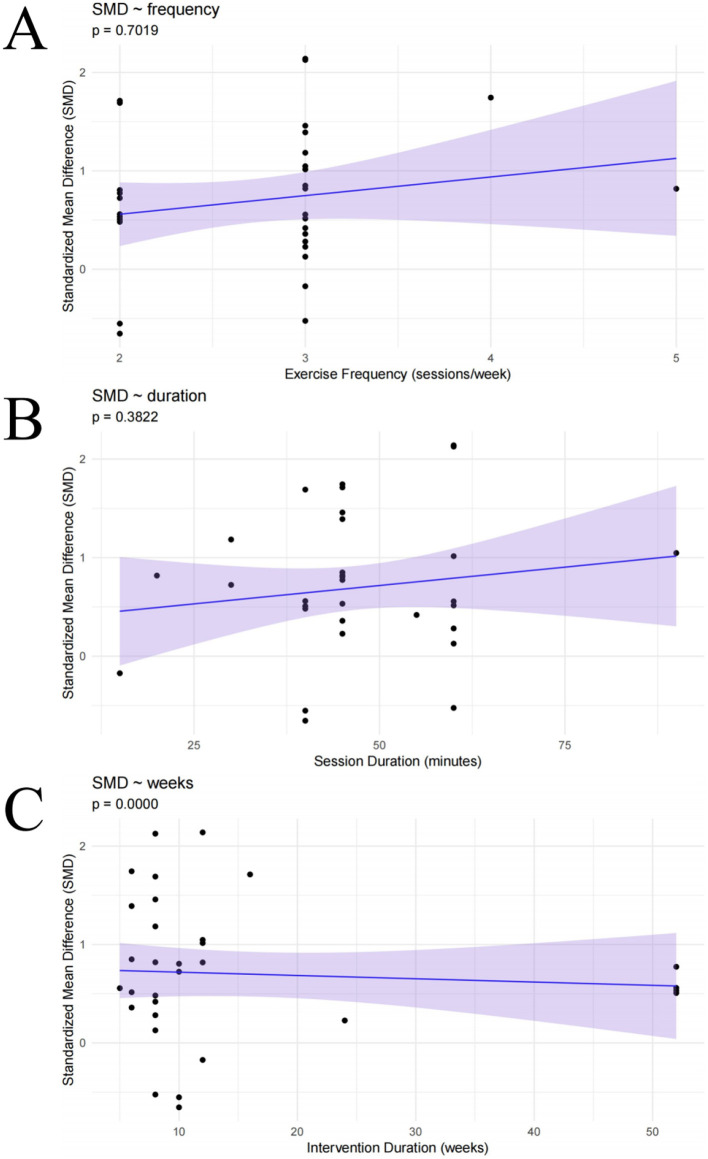
Meta-regression analyses: **(A)** intervention frequency; **(B)** session length; **(C)** intervention duration.

To explore potential sources of the moderate heterogeneity observed in the meta-analysis (*I*^2^ = 62.4%), a covariate-based meta-regression analysis was conducted (Figure 12). The results indicated a significant negative association between intervention duration and effect size (*p* < 0.001), suggesting that studies with shorter intervention periods tended to report larger intervention effects. In contrast, no significant linear associations were observed for intervention frequency (*p* = 0.70) or session length (*p* = 0.38). These findings suggest that variability in intervention duration may partially account for the inconsistencies in the overall effect estimates.

#### Sensitivity analysis

A leave-one-out sensitivity analysis was performed to examine the robustness of the meta-analytic findings (Supplementary material B). The re-estimated effect sizes, obtained after sequentially excluding each individual study, all fell within the 95% confidence interval of the original meta-analysis (approximately 0.45–0.92). The magnitude of change in effect sizes was minimal, and no single study was found to exert a substantial influence on the overall pooled effect. These results indicate that the estimated effect of exercise interventions on balance performance in children and adolescents with intellectual disabilities is robust and stable.

## Discussion

### Synthesis of evidence

This study systematically evaluated the effects of exercise interventions on balance performance in children and adolescents with intellectual disabilities and, for the first time, incorporated metabolic equivalents (MET·min/week) to construct a dose–response model. This approach enabled quantification of the non-linear relationship between exercise dosage and intervention efficacy, providing an evidence-based reference for the development of individualized exercise prescriptions.

The findings demonstrated that exercise interventions significantly improved balance performance in this population, with statistically significant differences across dosage levels, confirming the presence of a non-linear dose–response relationship. Notably, substantial improvements in balance were achieved within the moderate-dose range, suggesting that high-intensity or high-volume interventions are not necessarily required to produce meaningful benefits.

Dose–response modeling further indicated that the peak effect on balance performance occurred at approximately 717 MET·min/week (Hedges' *g* = 0.76). Notably, the World Health Organization (39) recommends that children and adolescents engage in an average of 60 min of moderate-to-vigorous physical activity per day (40), equivalent to roughly 1,680 MET·min/week. The optimal dose identified in this study is therefore about 43% of the WHO target, suggesting that, in children and adolescents with intellectual disabilities, a moderate exercise dose may achieve a more favorable balance between efficacy, safety, and adherence. This finding aligns with prior studies. For example, Ma et al. (41) reported that low-to-moderate intensity, regular training produced significant improvements in gait and postural control in children with ID within a short intervention period. Peters-Scheffer et al. (42) emphasized the superior long-term adherence and sustainability of moderate-intensity programs in early interventions. Similarly, randomized controlled trials in older adults with mild cognitive impairment and balance training programs in children with autism spectrum disorder have highlighted the safety, engagement, and stability of moderate-intensity exercise effects (38, 43). Even among healthy adolescents, moderate-dose activity can significantly improve balance and core stability within 6–8 weeks (44), consistent with the optimal dose range observed in this study. By contrast, the findings differ from those of Wouters et al. (45), who reported an advantage for moderate-to-vigorous activity in children and adolescents with moderate-to-severe ID. This discrepancy may be attributable to (1) differences in participant characteristics, as Wouters et al. (45) focused on individuals with more severe impairments, whose daily activity levels and exercise tolerance were generally lower, whereas the present study included primarily mild-to-moderate cases with greater capacity for structured training; and (2) differences in intervention design, as Wouters et al. (45) employed a cross-sectional observational approach linking higher-intensity activity with overall activity levels, whereas the present study synthesized structured, multicomponent interventions tailored to varying ability levels.

The subgroup analyses further supported the robustness and broad applicability of moderate-dose interventions. Among different exercise prescription combinations, structured programs delivered more than three times per week, lasting over 60 min per session, and conducted for fewer than 8 weeks achieved the largest effect sizes. Although some studies employed higher total doses or longer intervention periods, the intervention effects did not continue to increase with exercise volume. This phenomenon may reflect the cognitive, motor execution, and exercise tolerance limitations in children with ID (46), as higher loads can lead to fatigue, reduced attention, or decreased adherence, ultimately attenuating benefits or increasing injury risk (47).

Meta-regression analysis identified intervention duration as a significant moderator, with shorter programs associated with larger effect sizes, partially explaining the overall heterogeneity. In contrast, frequency and session length did not emerge as significant independent predictors, indicating limited explanatory power when considered in isolation.

Based on the totality of evidence, we recommend prioritizing structured moderate-dose exercise programs—approximately 500–800 MET·min/week, delivered more than three times per week, lasting over 60 min per session, and implemented for fewer than eight weeks—in interventions for children and adolescents with intellectual disabilities. Such programs balance efficacy, safety, and feasibility, are conducive to sustained participation, and provide empirically grounded guidance for the development of individualized, evidence-based exercise prescriptions in this special population.

### Clinical implications of the dose–response analysis

This study underscores the pivotal role of exercise in improving balance performance among children and adolescents with intellectual disabilities, demonstrating that even moderate-dose interventions can yield substantial benefits. In practical settings, individuals with intellectual disabilities often face cognitive limitations or motor execution impairments that hinder their ability to adhere to conventional high-intensity or high-volume training regimens. Under such circumstances, moderate-dose interventions become particularly valuable, as they not only ensure meaningful improvements but also allow participants to gradually adapt to training within their tolerable range.

Progressive, moderate-intensity programs can enhance balance and postural control without imposing excessive cognitive demands or inducing fatigue, thereby supporting sustained engagement. As emphasized by Manfredini et al. (48), low-to-moderate intensity, continuous interventions are more sustainable and safer for special populations. The present findings further reinforce the necessity of tailoring exercise prescriptions to individual needs, taking into account factors such as cognitive level, personal interests, and educational environment (49).

Such a patient-centered approach may foster a shift toward more personalized practice in rehabilitation and public health interventions (50). While this study confirms the beneficial effects of exercise interventions, notable research gaps remain. To better meet clinical needs, future studies should explore the efficacy of different intervention modalities under varying moderating conditions. Clarifying the relationship between exercise dosage and intervention outcomes is therefore of critical importance for optimizing intervention strategies. The insights from this study can provide clinicians and researchers in the rehabilitation field with practical, evidence-based methodological guidance.

### Strengths and limitations

This study is the first dose–response meta-analysis to systematically evaluate the effects of exercise interventions on balance performance in children and adolescents with intellectual disabilities, innovatively introducing metabolic equivalents (MET·min/week) to quantify intervention dosage and construct a non-linear dose–response model. A comprehensive search of five major databases was performed, with study selection conducted independently by two reviewers. Subgroup and sensitivity analyses were employed to ensure the stability and interpretability of the results. The findings provide a methodological foundation for developing individualized exercise prescriptions.

Nevertheless, several limitations should be acknowledged. Potential language and geographic biases may exist, some subgroups had relatively small sample sizes, and long-term follow-up data were lacking. Future studies should standardize the reporting of intervention parameters, expand sample sizes and geographic representation, and strengthen data collection in low- and middle-income countries to enhance the generalizability and practical relevance of the evidence.

## Conclusion

This study demonstrates that exercise interventions can significantly improve balance performance in children and adolescents with intellectual disabilities. Compared with conventional high-dose interventions, a moderate exercise dose of approximately 717 MET·min/week yielded more favorable outcomes. Intervention protocols incorporating at least three sessions per week, a session duration exceeding 60 min, and a total intervention period of no more than 8 weeks are recommended.

However, limitations remain, including small sample sizes, short follow-up periods, and the presence of moderate heterogeneity, which may restrict the generalizability and applicability of the findings. Future multicenter studies with larger samples, diverse severity levels, and varied intervention modalities, combined with long-term follow-up, are warranted to provide robust evidence for the individualized optimization of exercise prescriptions and the advancement of precision health strategies in this population.

## Data Availability

The original contributions presented in the study are included in the article/Supplementary material, further inquiries can be directed to the corresponding author.
