# Supplementary material for: Dose–response relationship of physical exercise interventions for balance performance in children and adolescents with intellectual disabilities: a systematic review and meta-analysis
Source: Front Public Health. 2025 Sep 25;13:1686892. doi: 10.3389/fpubh.2025.1686892 (PMC12507773; doi:10.3389/fpubh.2025.1686892)
Supplement: Supplementary file 1 [file Table_1.docx]

Section A

| **Detailed Search Strategies for Each Database** | |
| --- | --- |
| PubMed search: July 25, 2025 |  |
| (("Intellectual Disability"[Mesh] OR "Developmental Disabilities"[Mesh] OR "Mental Retardation"[Mesh] OR "Cognitive Dysfunction"[Mesh] OR "Learning Disabilities"[Mesh]OR "intellectual disability" OR "mental retardation" OR "cognitive impairment" OR "developmental delay" OR "developmental disability" OR "learning difficulty")AND("Exercise"[Mesh] OR "Exercise Therapy"[Mesh] OR "Motor Activity"[Mesh] OR "Physical Fitness"[Mesh] OR "Sports"[Mesh] OR "Rehabilitation"[Mesh]OR exercise OR "physical activity" OR "motor training" OR "aerobic training" OR "resistance training" OR "balance training" OR "flexibility training" OR "fitness program" OR "movement therapy" OR "motor learning")AND("Child"[Mesh] OR "Adolescent"[Mesh] OR "Pediatrics"[Mesh]OR child* OR children OR adolescent* OR teen* OR youth OR "school-age" OR "young people")AND("Postural Balance"[Mesh] OR "Postural Control"[Mesh] OR "Equilibrium"[Mesh] OR "Motor Skills"[Mesh]OR balance OR "postural control" OR "postural stability" OR "static balance" OR "dynamic balance" OR "Berg Balance Scale" OR BBS OR "center of pressure" OR "one leg stance" OR sway OR "body equilibrium" OR "gross motor function" OR "balance ability" OR "stability")) | 833 |
| Web of Science search: July 25, 2025 |  |
| TS=("intellectual disability" OR "developmental disabilities" OR "mental retardation" OR "cognitive dysfunction" OR "learning disabilities" OR "cognitive impairment" OR "developmental delay" OR "developmental disability" OR "learning difficulty") AND TS=("exercise" OR "exercise therapy" OR "motor activity" OR "physical fitness" OR "sports" OR "rehabilitation" OR "physical activity" OR "motor training" OR "aerobic training" OR "resistance training" OR "balance training" OR "flexibility training" OR "fitness program" OR "movement therapy" OR "motor learning") AND TS=("child" OR "adolescent" OR "pediatrics" OR child* OR children OR adolescent* OR teen* OR youth OR "school-age" OR "young people") AND TS=("postural balance" OR "postural control" OR "equilibrium" OR "motor skills" OR balance OR "postural stability" OR "static balance" OR "dynamic balance" OR "Berg Balance Scale" OR BBS OR "center of pressure" OR "one leg stance" OR sway OR "body equilibrium" OR "gross motor function" OR "balance ability" OR stability) | 825 |
| Cochrane Library search: July 25, 2025 |  |
| #1 "intellectual disability":ti,ab,kw OR "developmental disabilities":ti,ab,kw OR "mental retardation":ti,ab,kw OR "cognitive dysfunction":ti,ab,kw OR "learning disabilities":ti,ab,kw OR "cognitive impairment":ti,ab,kw OR "developmental delay":ti,ab,kw OR "developmental disability":ti,ab,kw OR "learning difficulty":ti,ab,kw  #2 "exercise":ti,ab,kw OR "exercise therapy":ti,ab,kw OR "motor activity":ti,ab,kw OR "physical fitness":ti,ab,kw OR "sports":ti,ab,kw OR "rehabilitation":ti,ab,kw OR "physical activity":ti,ab,kw OR "motor training":ti,ab,kw OR "aerobic training":ti,ab,kw OR "resistance training":ti,ab,kw OR "balance training":ti,ab,kw OR "flexibility training":ti,ab,kw OR "fitness program":ti,ab,kw OR "movement therapy":ti,ab,kw OR "motor learning":ti,ab,kw  #3 "child":ti,ab,kw OR "adolescent":ti,ab,kw OR "pediatrics":ti,ab,kw OR child*:ti,ab,kw OR children:ti,ab,kw OR adolescent*:ti,ab,kw OR teen*:ti,ab,kw OR youth:ti,ab,kw OR "school-age":ti,ab,kw OR "young people":ti,ab,kw  #4 "postural balance":ti,ab,kw OR "postural control":ti,ab,kw OR "equilibrium":ti,ab,kw OR "motor skills":ti,ab,kw OR balance:ti,ab,kw OR "postural stability":ti,ab,kw OR "static balance":ti,ab,kw OR "dynamic balance":ti,ab,kw OR "Berg Balance Scale":ti,ab,kw OR BBS:ti,ab,kw OR "center of pressure":ti,ab,kw OR "one leg stance":ti,ab,kw OR sway:ti,ab,kw OR "body equilibrium":ti,ab,kw OR "gross motor function":ti,ab,kw OR "balance ability":ti,ab,kw OR stability:ti,ab,kw  #5 #1 AND #2 AND #3 AND #4 | 179 |
| Embase search: July 25, 2025 |  |
| ('intellectual disability':ti,ab,kw OR 'developmental disabilities':ti,ab,kw OR 'mental retardation':ti,ab,kw OR 'cognitive dysfunction':ti,ab,kw OR 'learning disabilities':ti,ab,kw OR 'cognitive impairment':ti,ab,kw OR 'developmental delay':ti,ab,kw OR 'developmental disability':ti,ab,kw OR 'learning difficulty':ti,ab,kw) AND ('exercise':ti,ab,kw OR 'exercise therapy':ti,ab,kw OR 'motor activity':ti,ab,kw OR 'physical fitness':ti,ab,kw OR 'sports':ti,ab,kw OR 'rehabilitation':ti,ab,kw OR 'physical activity':ti,ab,kw OR 'motor training':ti,ab,kw OR 'aerobic training':ti,ab,kw OR 'resistance training':ti,ab,kw OR 'balance training':ti,ab,kw OR 'flexibility training':ti,ab,kw OR 'fitness program':ti,ab,kw OR 'movement therapy':ti,ab,kw OR 'motor learning':ti,ab,kw) AND ('child':ti,ab,kw OR 'adolescent':ti,ab,kw OR 'pediatrics':ti,ab,kw OR child*:ti,ab,kw OR children:ti,ab,kw OR adolescent*:ti,ab,kw OR teen*:ti,ab,kw OR youth:ti,ab,kw OR 'school-age':ti,ab,kw OR 'young people':ti,ab,kw) AND ('postural balance':ti,ab,kw OR 'postural control':ti,ab,kw OR 'equilibrium':ti,ab,kw OR 'motor skills':ti,ab,kw OR balance:ti,ab,kw OR 'postural stability':ti,ab,kw OR 'static balance':ti,ab,kw OR 'dynamic balance':ti,ab,kw OR 'Berg Balance Scale':ti,ab,kw OR BBS:ti,ab,kw OR 'center of pressure':ti,ab,kw OR 'one leg stance':ti,ab,kw OR sway:ti,ab,kw OR 'body equilibrium':ti,ab,kw OR 'gross motor function':ti,ab,kw OR 'balance ability':ti,ab,kw OR stability:ti,ab,kw) | 343 |
| Scopus search: July 25, 2025 |  |
| TITLE-ABS-KEY("intellectual disability" OR "developmental disabilities" OR "mental retardation" OR "cognitive dysfunction" OR "learning disabilities" OR "cognitive impairment" OR "developmental delay" OR "developmental disability" OR "learning difficulty")  AND  TITLE-ABS-KEY("exercise" OR "exercise therapy" OR "motor activity" OR "physical fitness" OR "sports" OR "rehabilitation" OR "physical activity" OR "motor training" OR "aerobic training" OR "resistance training" OR "balance training" OR "flexibility training" OR "fitness program" OR "movement therapy" OR "motor learning")  AND  TITLE-ABS-KEY("child" OR "adolescent" OR "pediatrics" OR child* OR children OR adolescent* OR teen* OR youth OR "school-age" OR "young people")  AND  TITLE-ABS-KEY("postural balance" OR "postural control" OR "equilibrium" OR "motor skills" OR balance OR "postural stability" OR "static balance" OR "dynamic balance" OR "Berg Balance Scale" OR BBS OR "center of pressure" OR "one leg stance" OR sway OR "body equilibrium" OR "gross motor function" OR "balance ability" OR stability) | 994 |

Section B


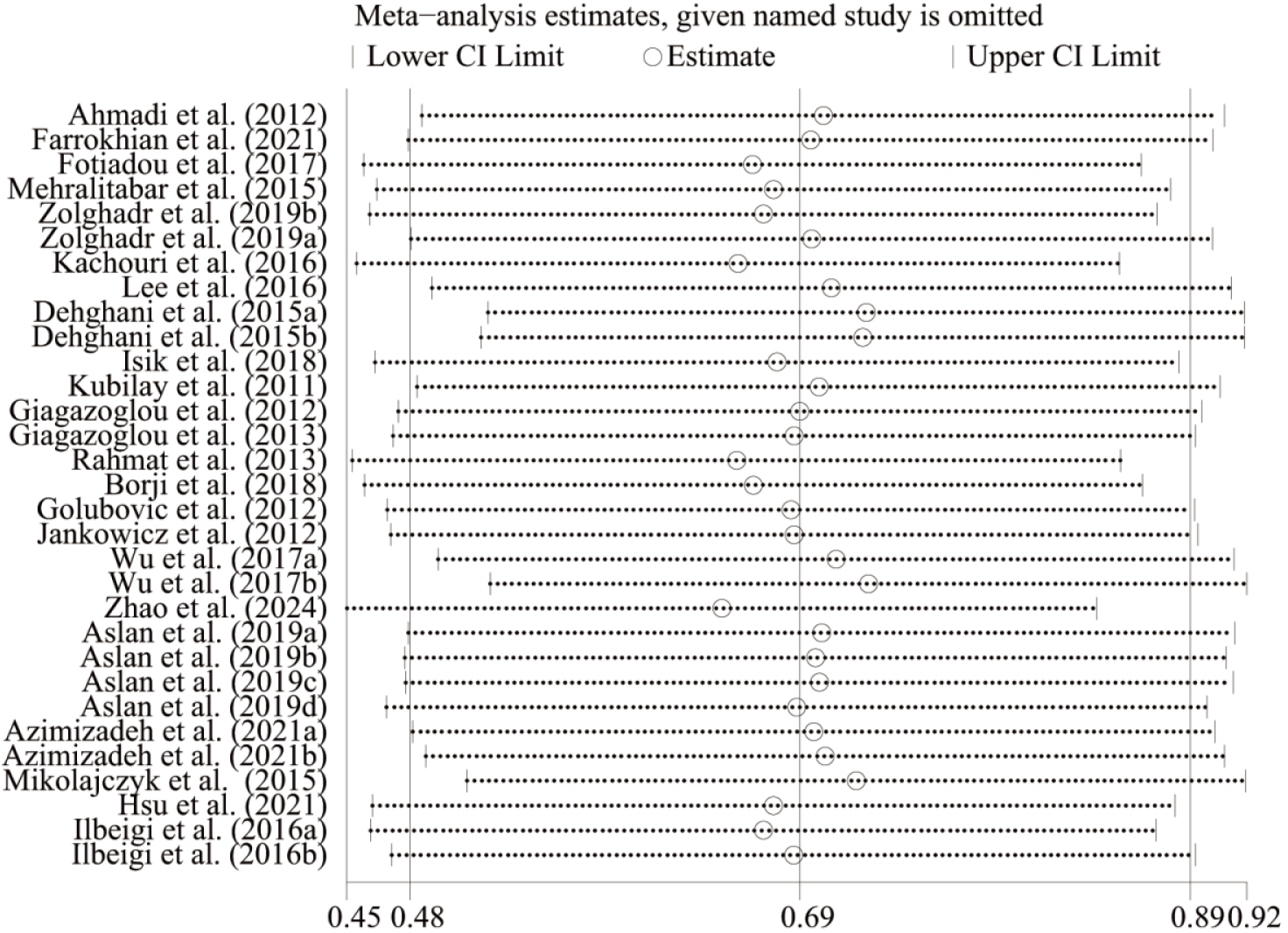


**Supplementary Figures 1**. Sensitivity analysis
